# Negative Pressure Wound Therapy as an Alternative to Flap Reconstruction in an Extensive 16.5 × 11 cm Pilonidal Sinus Excision: A Case Report

**DOI:** 10.7759/cureus.91064

**Published:** 2025-08-26

**Authors:** Ata Maden, Amram Kupietzky, Noemie Nalbandian, Ido Mizrahi

**Affiliations:** 1 General Surgery, Hadassah Medical Center, Jerusalem, ISR; 2 Wound and Stoma Care, Hadassah Medical Center, Jerusalem, ISR

**Keywords:** case report, negative pressure wound therapy, pilonidal sinus disease, secondary closure, surgical wound management, vacuum-assisted closure, wound healing

## Abstract

Pilonidal sinus disease (PSD) is a chronic condition often requiring surgical excision, particularly in cases of recurrent inflammation. Large post-excisional defects lack universally accepted management guidelines, and closure technique selection typically depends on the operating surgeon's preference and institutional resources. We report the case of a 21-year-old healthy man with a 2.5-year history of recurrent PSD, requiring wide excision that resulted in a markedly large 16.5 × 11 cm defect with a depth of 2.8 cm. In light of the wound's size and the intraoperative identification of active infection with surrounding inflammation, negative pressure wound therapy (NPWT) was selected as the most appropriate technique for secondary closure.

NPWT was initiated during postoperative hospitalization and continued for 32 days, followed by conventional wound care. Healing progression was documented with serial clinical and photographic documentation over 90 days, demonstrating progressive wound contraction and complete epithelialization by secondary intention without complications. Further follow‑up at six months, one year, and two years confirmed sustained healing without recurrence, and the patient reported high satisfaction with both functional and aesthetic outcomes.

This case highlights NPWT as a feasible and effective secondary closure method for extensive PSD excisions. It may be a surgically less invasive alternative to flap reconstruction in selected patients, offering simplified surgical management, acceptable morbidity, and potentially favorable long-term outcomes, albeit with a longer wound management period.

## Introduction

Pilonidal sinus disease (PSD) is a chronic inflammatory condition affecting the sacrococcygeal region. Early descriptions proposed congenital origins, but the disease is now recognized as an acquired disorder arising from hair penetration into the natal cleft, leading to a foreign body reaction, chronic inflammation, and sinus formation [1,2]. Hair, shear forces driving the hair into the tissue, and the vulnerability of the natal cleft skin constitute the essential etiologic factors [3]. Recent population‑based studies show that the incidence of PSD is rising, reaching 39.6-56 cases per 100,000 person‑years, and the male‑to‑female ratio remains approximately 3.1:1, with a peak incidence in early adulthood [4,5]. Several modifiable and non‑modifiable risk factors have been proposed, including male sex, positive family history, obesity, sedentary lifestyle, and hirsutism [4-6]. These factors can also contribute to chronicity and recurrence, and patients often experience pain, recurrent abscesses, restricted mobility, and psychological distress [6].

Management strategies for PSD vary widely, reflecting both differing institutional practices and individual surgeon preference, alongside a lack of consensus on optimal surgical techniques [7]. This variability is further demonstrated in national surveys, such as a Dutch study observing substantial heterogeneity across modalities including excision, flaps, and minimally invasive interventions [8].

Non‑operative measures include hair removal and hygiene, while surgical options range from simple incision and drainage to wide excision with closure techniques. Primary midline closure has historically shown high recurrence rates. Multiple studies, including a meta‑analysis of randomized trials, have shown recurrence rising from approximately 7% at 24 months to as high as 67.9% at 240 months, whereas off‑midline advancement or rotational flaps consistently maintain recurrence between 0.2% and 5.2% over 12-60 months [9]. Off-midline techniques such as Limberg and Karydakis flaps, as well as the cleft lift procedure, are generally preferred for large defects because they flatten the natal cleft and move the scar laterally. However, flap surgery involves more extensive tissue manipulation, longer operative time, and a higher risk of donor site complications, which may not be preferable in every clinical scenario.

Negative pressure wound therapy (NPWT) has gained attention as an alternative to flap reconstruction. By applying sub‑atmospheric pressure, NPWT removes exudate, reduces edema, decreases bacterial load, and stimulates granulation tissue [10]. A randomized trial comparing NPWT with standard open wound care after PSD excision found that NPWT reduced wound size more rapidly (wound size ratio 0.30 vs. 0.57 at two weeks) but did not significantly shorten median healing time or time to resume daily activities [10]. A recent prospective cohort study combining sinus excision with NPWT reported shorter operation time (46.9 vs. 58.7 minutes), reduced hospital stay (19.2 vs. 21.8 days), and faster return to normal activities (21.2 vs. 23.8 days) compared with primary closure [11]. A systematic review and meta‑analysis of 10 studies (609 participants) found that NPWT may shorten median healing time by 9-34 days, lower recurrence (4% vs. 11%; odds ratio: 0.39), and reduce pain, although the quality of evidence was low [12].

We report the case of a 21-year-old man with recurrent PSD for 2.5 years, presenting with an exceptionally large disease area (16.5 × 11 × 2.8 cm), successfully treated with wide excision and NPWT, achieving high patient satisfaction. This case contributes to the growing body of evidence supporting NPWT as a viable alternative to flap reconstruction, particularly for extensive defects, and highlights its potential for promoting complete healing without added surgical morbidity.

## Case presentation

A 21-year-old otherwise healthy male (BMI 23.8) university student presented with a 2.5-year history of recurrent PSD. Initial treatments at another institution included multiple incision and drainage procedures. The patient was advised to undergo definitive surgical management but did not attend relevant follow-ups. He presented to our institution with another abscess in a progressively enlarging disease area. The abscess was drained in the operating room, with multiple drains placed. After a course of oral antibiotics (amoxicillin-clavulanate), the drains were removed during clinical follow-up, and definitive surgery was planned two months later.

The patient had no comorbidities, and preoperative evaluation was unremarkable, requiring no further optimization.

**Figure 1:**
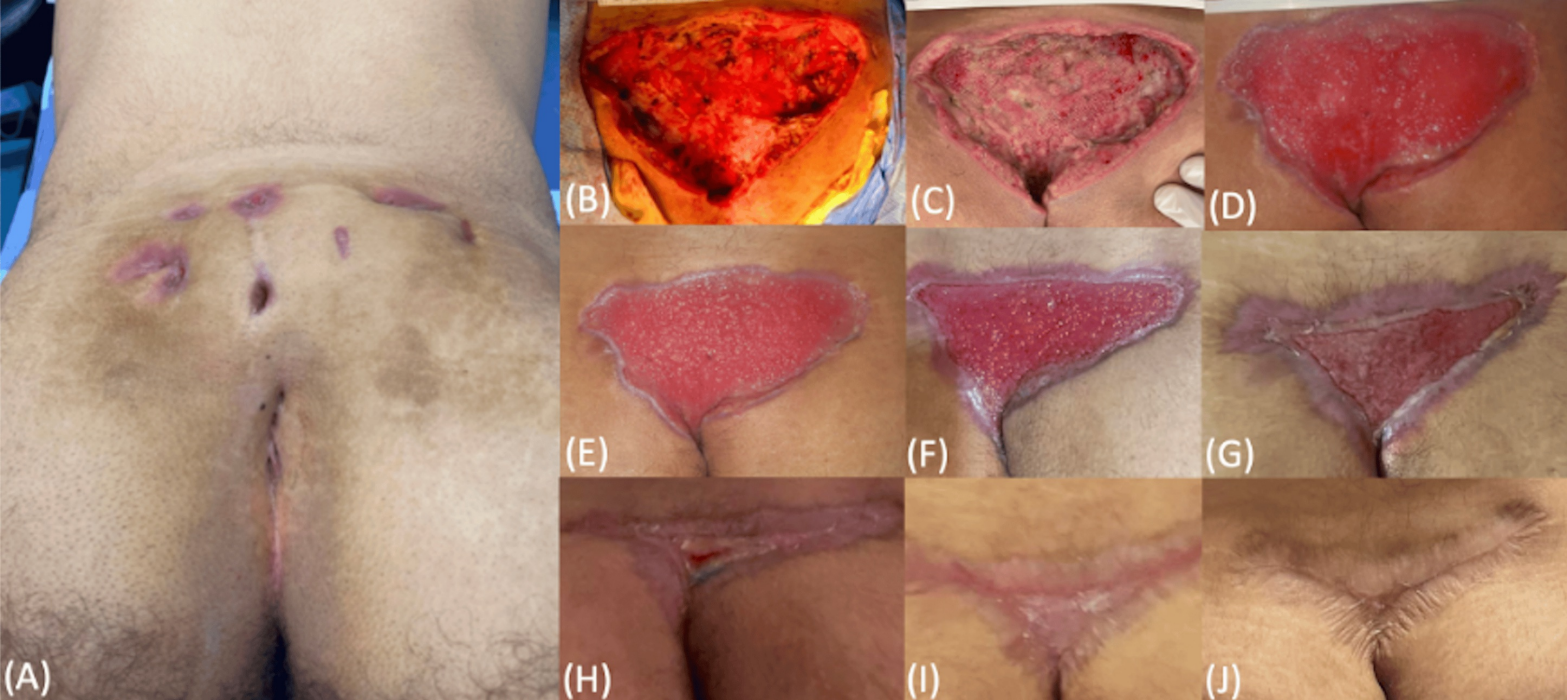
Photographic follow-up of wound healing. (A) Preoperative presentation. (B) Post-excision view (day 0). (C) Seven days post-op. (D) Twenty-five days post-op. (E) Thirty-two days post-op. (F) Forty-two days post-op. (G) Fifty-nine days post-op. (H) Seventy-eight days post-op. (I) Ninety days post-op. (J) Six months post-op.

Upon elective admission, physical examination revealed an extensive PSD with multiple external openings, the farthest being 12.5 cm from the primary lesion (Figure 1A). Indurated and fluctuant areas were palpated. Under spinal anesthesia, all external openings were probed, confirming fistulous connections to the primary sinus. A wide excision was performed down to the fascia with negative margins. Residual pus was drained, and specimens were sent for pathological and microbiological examination. Primary closure, flap formation, and skin graft techniques were avoided due to the wound size and active infection with surrounding inflammation. The procedure was performed by a senior colorectal surgeon at our tertiary university hospital.

Post-excision, the wound measured 16.5 × 11 cm with a maximum depth of 2.8 cm (Figure 1B). After meticulous hemostasis and irrigation with povidone-iodine and saline, the surgical bed was initially packed with saline-soaked gauze. The patient received amoxicillin-clavulanate for the intraoperatively observed active infection along with routine wound care. Given the wound's primarily cranial extension from the sacrococcygeal region and the absence of perianal involvement, NPWT was deemed appropriate. A wound care nurse applied NPWT, and the patient was discharged for outpatient follow-up. Blood tests during hospitalization were unremarkable and consistent with the clinical and intraoperative findings. No imaging was performed.

NPWT was continued for one month, after which conventional wound care was maintained with regular cleaning, inspection, and hair removal around the surgical site. Wound dimensions and healing progression were documented throughout follow-up with serial outpatient visits, as summarized in Figure 1 and Table 1.

**Table 1:** Follow-up of wound dimensions and healing progress, with references to Figure 1. NPWT: negative pressure wound therapy

Day	Figure reference	Wound size (cm)	Depth (cm)	Notes
0	Figure 1B	16.5 × 11	2.8	Large open wound post-excision
7	Figure 1C	16 × 10	2.5	Early granulation, healthy wound base
25	Figure 1D	13 × 8.5	2.0	Dense granulation, peripheral contraction
32	Figure 1E	11.5 × 6.5	1.8	Epithelialization, NPWT discontinued
42	Figure 1F	9 × 5	1.2	Noticeable edge advancement
59	Figure 1G	7 × 3.5	0.7	Contraction continues, depth shallowing
78	Figure 1H	5 × 2	0.3	Superficial, closure nearing completion
90	Figure 1I	3 × 1.5	0.2	Final epithelialization, wound fully closed

At 90 days, follow-up was discontinued. Six months later, physical examination at our outpatient clinic revealed complete healing with no recurrence or residual discomfort (Figure 1J). From then on, a phone call follow-up was done at one year and two years after the surgery with no change in these findings. Throughout the follow-up, the patient consistently reported high satisfaction with the aesthetic and functional outcome. He demonstrated full adherence to NPWT use and outpatient follow-up visits, including wound care instructions and hair removal. No deviations from the planned treatment occurred.

## Discussion

PSD presents a significant clinical challenge due to the complexities associated with wound management post-excision and its propensity for recurrence. Traditional treatment modalities have been associated with prolonged healing times and high recurrence rates, prompting the exploration of alternative approaches to improve outcomes. Healing times for open wounds may extend up to two to three months, while primary midline closure is associated with high long‑term recurrence rates [9,13]. Secondary healing techniques, particularly NPWT, are beneficial for large post-excisional defects, as they promote granulation and reduce infection risk. As previously described, NPWT supports healing through exudate removal, improved perfusion, and reduced bacterial burden.

According to recent surgical literature, wide excision of extensive PSD typically requires flap coverage, especially for large defects. Techniques such as Limberg, Karydakis, and cleft lift procedures are preferred in order to allow tension‑free closure and to lower recurrence [14]. While primary midline closure may offer faster initial healing, it carries higher rates of recurrence and surgical site infection (up to 24%) compared to off-midline flap techniques [15]. Flap-based closures reduce recurrence by positioning the scar off the midline, and while being generally effective, they may involve longer operative times and donor site morbidity, particularly in less specialized settings [9,14]. NPWT offers an alternative with progressive wound contraction and minimal surgical complexity without additional morbidity. Notably, a retrospective cohort study by Hannan et al. found that wide excision followed by NPWT was associated with low complication rates, short hospital stays, and very low recurrence, supporting its feasibility as an effective option in selected cases [16].

There is a growing literature underlining NPWT's role in significantly accelerating wound closure in extensive PSD cases. A recent prospective study investigated the use of NPWT vs. standard care following a wide local excision of complex or infected PSD and found a marked reduction in median wound closure time (59.2 vs. 75.3 days) [17].

While NPWT offers clear clinical benefits, its impact on patient quality of life and the overall strain on healthcare resources remains an important consideration. Patients treated with NPWT often report less pain and greater mobility compared to those receiving traditional packing dressings [12]. Portable NPWT systems, with their discreet design and ease of use, have been linked to improved compliance and overall satisfaction [18]. NPWT devices and supplies can be expensive; for example, one analysis found the cost per patient treated with NPWT was approximately $142 higher than with standard dressings [19]. However, this direct cost may be offset by downstream savings if NPWT reduces complications or facilitates healing in cases where traditional closure techniques are not feasible. In a recent study on wound care, NPWT significantly decreased hospital length of stay and infection rates without increasing total treatment cost [20]. The cost impact of NPWT was not formally assessed in our case. Nevertheless, the potential increase in consumable use, device-related expenditures, and logistical burdens (particularly in outpatient settings) warrants deeper evaluation of its long-term cost-effectiveness.

This case contributes a descriptive account of successful secondary healing of a markedly large PSD wound using NPWT alone, in a setting where flap reconstruction was deemed not appropriate due to active infection. As the selected alternative, NPWT offered a practical solution that avoided donor-site morbidity and the need for complex tissue rearrangement. The size of the defect in our patient exceeded those reported in most studies evaluating NPWT, yet granulation and contraction progressed steadily, and epithelialization was complete by day 90. The patient maintained excellent adherence to NPWT and follow‑up appointments and achieved high satisfaction with cosmetic and functional outcomes.

Although this is a single case, it highlights that in carefully selected patients with extensive defects and good compliance, NPWT can be a viable alternative to flap closure, particularly in clinical scenarios where primary closure or flaps are either contraindicated due to local factors or considered undesirable based on intraoperative judgment. This approach may promote complete healing with acceptable morbidity, expanding the armamentarium for managing severe PSD. Each patient should be individually evaluated, and further studies can help clarify which subsets benefit most from NPWT, but our experience suggests that even very large and infected pilonidal wounds may be successfully managed with this technique.

## Conclusions

This case demonstrates that NPWT can be an effective and well-tolerated alternative to flap reconstruction following a wide excision of extensive PSD, particularly when primary closure or flap reconstruction is not feasible due to local wound size and conditions. In this case, with a defect of 16.5 × 11 cm and a depth of 2.8 cm, wound management assisted by NPWT facilitated complete epithelialization without complications or recurrence and resulted in high patient satisfaction.

While NPWT may help avoid more invasive closure techniques in selected cases, its role should be individualized based on intraoperative findings and patient-specific factors. Further comparative studies are warranted to evaluate long-term outcomes, cost-effectiveness, and recurrence rates relative to traditional reconstructive approaches in complex PSD cases with substantial defects.
